# Exploring motivations to be active among amputees: a phenomenological approach to leisure time physical activity

**DOI:** 10.1080/17482631.2022.2143053

**Published:** 2022-11-09

**Authors:** Sara H. Olsen, Elizabeth M. Aparicio, Paul T. Jaeger, Donna E. Howard

**Affiliations:** a Department of Behavioral and Community Health, University of Maryland at College Park, College Park, Maryland, USA; b College of Information Studies, University of Maryland at College Park, College Park, Maryland, USA

**Keywords:** Amputee, physical activity, motivation, self-determination theory, interpretative phenomenology

## Abstract

**Purpose:**

People with disabilities are less active and experience increased burden of disease compared to those without disabilities. Leisure time physical activity (LTPA) participation is strongly related to improved health in all populations. Despite this, research with amputees focuses heavily on prosthetic design and function, leaving amputees an under-represented population in LTPA and motivation research. This study explored the lived experiences of motivation to engage in LTPA among amputees.

**Method:**

Interpretative phenomenological analysis (IPA) approach, influenced by understandings of embodiment and Self-Determination Theory (SDT), was used. Six people with lower extremity, acquired amputations created a photo-diary of their motivations to be active and participated in two in-depth interviews each.

**Results:**

Study findings suggest motivations to be active among amputees are primarily influenced by personal relationships. Barriers, facilitators, and processes to be active provided insight into how LTPA participation may be affected by more than motivation. Embodiment was experienced as bodily integration of the prosthesis.

**Conclusions:**

There is evidence that the relationship between motivation and participation is not direct, but influenced by facilitators, barriers, and processes to be active. The results suggest strategies to increase activity among amputees by emphasizing LTPA with others, improving the built environment, and ensuring prosthesis fit.

## Introduction

The benefits of physical activity (PA) are well documented. Participation in PA has an inverse dose-response relationship with all-cause mortality and is an effective prevention of at least 25 chronic illnesses, including cardiovascular disease and depression (Arem et al., 2015; Dunn et al., 2001; Ekelund et al., 2019; Moore et al., 2012; Pedersen & Saltin, 2015; Warburton et al., 2006). Findings from a meta-analysis of 22 cohort studies with greater than 10,000 participants, which estimated the relationship of PA to all-cause mortality, showed a non-linear dose-response relationship with greatest health benefits in the transition from inactivity to low levels of PA (Woodcock et al., 2011). Leisure time physical activity (LTPA), activity done at one’s own discretion, provides greater health benefits and greater opportunities to meet PA guideline recommendations than do other forms of PA, such as those related to transportation or occupational activity (Holtermann et al., 2018; Tsenkova, 2017; Vuillemin et al., 2005).

Among people with disabilities (PwD) in the USA, 47% are sedentary, which is much higher than the 24.3% of those without disabilities who are sedentary (Centers for Disease Control and Prevention (CDC), National Center on Birth Defects and Developmental Disabilities, Division of Human Development and Disability, 2017). The majority (57%) of people with mobility-related disabilities, such as wheelchair users and amputees, are inactive (Centers for Disease Control and Prevention (CDC), National Center on Birth Defects and Developmental Disabilities, Division of Human Development and Disability, 2014). Barriers to LTPA for people with mobility impairments are found at the intrapersonal, interpersonal, and environmental levels. PwD experience barriers related to motivation, lack of social support, and physical barriers in the built or natural environment (Deans et al., 2012; Malone et al., 2012; Martin Ginis et al., 2012). However, PwD who engage in LTPA show improvement in physical and emotional health, and strengthened social ties. LTPA participation among PwD increases aerobic capacity, muscular power output, and functional performance (Hicks et al., 2011; Lui & Hui, 2009; Martin Ginis et al., 2012; Wilhite & Shank, 2009). Participation also improves a variety of factors associated with psychosocial well-being including increased coping skills, confidence, social connectedness, and decreased symptoms of depression (Martin Ginis et al., 2017; Nooijen et al., 2017; Prout & Porter, 2017).

Historically, research related to amputees focuses on rehabilitative PA, gait improvement, the biomechanics of prosthesis use, and the physiology of limb loss (Bragaru et al., 2011; Castro et al., 2018; Lai et al., 2017). Some emerging research, however, suggests self-efficacy, social support, attitude, and the environment (e.g., neighbourhood crime, transportation accessibility, climate) all influence participation in PA among lower extremity amputees, but those studies focus on participation outcomes and do not address motivation (Batten et al., 2019; Miller et al., 2019). Amputees participating in a study to understand broad experiences of life as an amputee have also described how barriers and facilitators are variable and fluctuate on a daily basis, affecting the planning of LTPA participation (Day et al., 2019).

A few quantitative studies among PwD have addressed motivation directly or motivation within a theoretical framework such as Self-Determination Theory (SDT), suggesting the opportunity for more exploratory work to understand how amputees experience motivation to be active (Lai et al., 2017; Perreault & Vallerand, 2007). This study offers a novel understanding of LTPA participation by explicitly addressing motivation through a theoretical approach. The basic psychological needs (BPN) constructs of competence and relatedness were both found to mediate the relationship between peer mentorship and study outcomes of affect and participation (Sweet et al., 2018). In another study using motivational constructs of SDT, comparison of wheelchair basketball players with and without disabilities showed no significant differences in motivation for sport (Perreault & Vallerand, 2007). Findings may indicate some transferability of SDT-related results in LTPA interventions to non-intervention populations including PwD.

**Figure 1.**
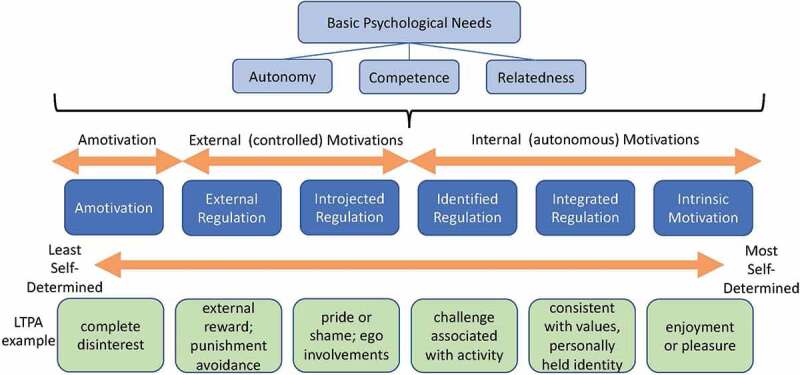
Self-Determination Theory (SDT) Model (adapted from Ryan & Deci, 2007).

This study integrated components of SDT as a framework for understanding and describing motivations (Figure 1; Gagné & Deci, 2014). SDT is a complex macro theory that incorporates five mini-theories (Deci & Ryan, 1985; Ryan & Deci, 2017). Three of these mini-theories informed this study. The first two mini-theories, Organismic Integration Theory (OIT) and Cognitive Evaluation Theory (CET), combine to create a continuum of motivation ranging from low internalization, also called controlled or amotivation, to the most autonomous motivation (intrinsic motivation), which represents full immersion in the activity with enjoyment of participation as its own reward (Vansteenkiste et al., 2010). Between amotivation and intrinsic motivation on the continuum are several type of extrinsic motivations, described as Regulations. Basic Psychological Needs Theory is the third mini-theory used to understand motivation in this study; it describes generalized BPN i.e., autonomy, competence, and relatedness. SDT suggests that when the environment supports fulfilment of needs for competence, relatedness, and autonomy, people will become more self-determined, which entails making decisions based on their own preferences and regulating their own actions.

Recognizing amputees are unique among PwD in their uses of assistive devices, this study aimed to incorporate Maurice Merleau-Ponty’s concepts of embodied phenomenology into the data collection and analysis. Merleau-Ponty argued that humans interact with their world through both thought and body, making meaning both somatically and cognitively, with neither way of knowing holding primacy (Crossley, 2012). Prostheses become a part of the body for amputees; literally, in the case of Intraosseous Transcutaneous Amputation Prostheses. The body interacts with the socket and attached components as a way of interacting with the environment and transmitting somatic knowledge. Meaning derived from and embedded in thoughts, feelings, and bodily (re)actions for amputees, therefore, may be different than for others (Murray, 2008; Wilson, 2015). Data collection incorporated embodied reactions and understandings to better understand amputees lived experiences with motivations to be active.

### Aim

This study aimed to explore the embodied meanings and lived experiences of motivation to engage in LTPA among amputees through photo-diaries and in-depth interviews.

## Method

### Design

Recognizing that PwD experience life wholly differently—and not simply as a disrupted or modified experience of nondisabled counterparts—we were interested in the phenomenon of motivation to be active as experienced by amputees, specifically unilateral lower limb amputees (Martiny, 2015). Interpretative phenomenological analysis (IPA) methods were used to investigate, in detail, the significance of LTPA motivation, barriers, facilitators, and bodily reactions to those events and how amputees make sense of their experiences (J. Smith et al., 2009). IPA was chosen to elucidate the active meaning-making individuals with unilateral amputation go through to understand their interactions with the world around them and their subjective reaction and interpretations of those events (Roșca et al., 2021). The process requires both the participants and the researcher to engage in reflection, working through the cognitive, emotional, and embodied understanding of the experience’s impact on the participants’ lives. To capture the experiences of LTPA, participants created photo-diaries and the technique of photo-elicitation was incorporated within the in-depth interviews (Burke, 2005). Because of COVID-19 restrictions on physical proximity, photo-elicitation was used to allow the researchers to experience the events with the participants through the participants own stories and interpretations of their experiences of motivation. During the second interview, participants were asked questions to try to take them back to the moment they took the picture e.g., “Think back to the moment you took this photo. Tell me about the sounds and smells in the area.” (Probes: normal sounds and smells, do they make you feel a certain way, can you hear/smell them now).

### Participants

Accepted phenomenological research practices vary in terms of recommended and/or appropriate sample sizes but maintain the emphasis on small numbers to ensure qualitative rigour and depth of analysis (Creswell, 1998; Morse, 2003; Onwuegbuzie & Collins, 2007). Recommended sample sizes vary greatly e.g., 3–10 participants (Dukes, 1984; Reid et al., 2005), 5–25 participants (Polkinghorne, 1989), 2–10 participants (Groenewald, 2004). Twelve people responded to a phone screener with eight meeting all inclusion criteria for the study. Those eight amputees completed consent forms and at least one interview; only six of whom chose to complete the photo-diary and both interviews (Table I). Recruitment was conducted through organizations and individuals trusted by, and with regular access to, the community of amputees in the USA. The primary partner organization supporting recruitment was the Amputee Coalition (AC). They are the leading advocacy organization for those living with limb loss. AC advertised the study through listserv emails, social media posts, mailings, and peer support group leaders. Participant inclusion criteria ensured an adult sample with similar characteristics such that a intensive interpretation of similar experiences could be conducted (Roșca et al., 2021): being a single limb amputee, amputation at or above the ankle, aged 18 through 65 years, with access to the internet for Zoom^TM^ meetings, and the ability to take digital photographs. Exclusion criteria included participation in a previously conducted app-based motivation intervention by authors, or those currently under the care of a physician for anything other than routine purposes. All participants were lower extremity amputees whose amputation was acquired.

**Table I.** Participant characteristics (n = 6).

Participant	Characteristics
Summer	Woman
	Age: 37
	Years living with amputation: 21
	Traumatic amputation
	Uses multiple mobility devices
Michael	Man
	Age: 62
	Years living with amputation: 9
	Bacteria-associated amputation
	Uses prosthesis
Roslyn	Woman
	Age: 42
	Years living with amputation: 4.5
	Post-surgical infection-related amputation
	Uses multiple mobility devices
Dawn	Woman
	Age: 57
	Years living with amputation: 4
	Traumatic amputation
	Uses multiple mobility devices
Kristin	Woman
	Age: 59
	Years living with amputation: 19
	Traumatic amputation
	Uses multiple mobility devices
Tiffany	Woman
	Age: 61
	Years living with amputation: 6.5
	Cancer-related amputation
	Uses multiple mobility devices

Potential participants were directed to the research team for a complete description of the study, time commitment, and incentives. Potential participants underwent a phone screening to verify interested individuals met inclusion criteria. The phone screening provided a full explanation of the study. Participants were offered e-gift cards in the amount of $25 for the first interview and $35 for the second interview as incentives for participating in the study and to compensate them for their time. The individuals’ inclusion in or decision (not) to participate in the study was not shared with the recruitment partners.

### Data collection

Data were collected through two in-depth, semi-structured interviews and included a participant developed photo-diary related to LTPA. The open-ended nature of the interview questions provided the participants with the freedom to use their own words and the researcher to allow the interview to go where the participant took it, modifying the questions as the interviews progressed (Daly, 2007; Miles & Gilbert, 2005). Each interview lasted 45–75 minutes. The interview guides were designed to explore 1) current motivations for LTPA, especially as they aligned with or contradicted SDT concepts; 2) past experiences with LTPA; and 3) attitudes and embodied understandings related to LTPA as an amputee. Interviewers defined LTPA for the participants as “activity you choose to do that is not related to your job, maintaining your home, or transportation. These can be hobbies like gardening or yoga, or fitness activities like walking or lifting, or more traditional sports like golf or tennis.” The interview guides were pilot tested with two amputees known to the first author and not drawn from the pool of eligible participants. The first interview explored participants’ experiences and meanings of motivation to be active. Some examples of questions include: Thinking back at least 2 years, what sort of leisure time physical activities have interested you in the past? (Probes: as a child/adolescent, before amputation if acquired, pre-COVID-19, school activities); and think about other things you do outside of work. What motivates you to participate in those activities? (Probes: fun, easy, no pain, can do at home, friends also participate, risk level)

Between the first and the second interview, participants were asked to create a photo-diary of anything that would help them describe their experiences related to motivation to be active. Participants were asked to take 4–8 pictures of any activity they regularly participated in or considered participating in. The description of the photo-diary made clear the diary should capture experiences around motivation to be active, with no obligation to have ever participated in the activity or to participate in the activity during this study. Participants were asked to represent their process to get ready for, participate in, and recover from their chosen activity or to document things that influenced their decision not to participate. Once participants had taken photographs and provided them to the first author, arrangements were made for the second interview to discuss the photos. Participants determined the activity, the subject of the images, and which images to talk about during the second interview. Embodied research necessarily includes components of lived experiences within space and over time (Spatz, 2017). The second round of interviews were designed to elicit embodied experiences of being active. Accordingly, participants were asked not only to describe motivations and LTPA activities captured in their photo-diaries but to return to the moment through photo-elicitation interview techniques (Burke, 2005; Johnson-Glenberg & Megowan-Romanowicz, 2017; Magnat, 2011; Pink, 2011; Spatz, 2017).

Due to the ongoing COVID-19 pandemic, all interviews occurred via the video web app Zoom. The interviewer shared their screen for the second interview to allow both the participant and the interviewer to see the picture as questions were asked. This allowed participants to return to the moment the picture was taken, both visually and as guided through the interview questions (Pink, 2011). All interviews were audio recorded and subsequently transcribed verbatim for analysis. Participants chose pseudonyms before the recording began.

### Data analysis

Data were analysed from the perspective that the experience meant to be understood is the amputee’s motivation to participate in LTPA and the experiences that influence that motivation. To facilitate this process, we relied on the method outlined by J. Smith et al. (2009); (2022). The analysis process incorporated peer debriefings throughout and member reflections to improve trustworthiness and credibility of results. Peer debriefing is the process of engaging colleagues who hold impartial views of the study. The impartial peers critically review methodology, implementation, and data analysis procedures (Spall, 1998). For this study, seven peer debriefers were asked to provide their views of the study, field notes, and analysis. These peers were chosen as those who might be familiar with the population or the methods but were otherwise not connected to the research. Member reflections involved sharing the study’s findings with participants and providing opportunities for feedback and insight (Smith & McGannon, 2018). The six steps of IPA are:

1. Reading and re-reading. Verbatim transcriptions were read and reread to understand participant’s narrative descriptions of LTPA motivation as an amputee. This was immersive in nature and offered the opportunity for prolonged exposure to the data as is necessary in phenomenological research.
2. Initial noting. This step was exploratory in nature and the most detailed of the analytical steps. Annotations were categorized as descriptive, linguistic, and conceptual and were intended to focus on what is important to the participants (J. Smith et al., 2009). Descriptive annotations represented events, relationships, and values through the eyes of participants. Linguistic annotations paid particular attention to the words, phrases, and expressions—the specific use of language—participants use. Conceptual annotations began to interpret participants’ understandings and perceptions of their experiences.
3. Developing experiential statements. Experiential statements summarized individual participant stories, produced through integration of the data and theoretical assumptions brought into the process through the use of a SDT framework. Notes and transcripts were reviewed to map interrelationships of ideas, connections between exploratory notes, and to reduce the volume of detail.
4. Searching for connections across experiential statements within participant data. The experiential statements were reviewed for grouping within a single participant’s transcript. The experiential statements that clustered into a single cohesive idea were combined to form personal experiential themes. The transcripts were reviewed for oppositional relationships as well as complimentary ones, i.e., a participant may have expressed the importance of close relationships in their LTPA participation in one section and expressed the impact of stigma in another section. Both of these statements would have been included in personal experiential themes describing to relatedness, but would have been clustered very differently in this step to indicate different individual experiences with relatedness.
5. Moving to the next participant. Steps 1–4 were repeated for each participant. The transcripts were read, noted, and personal experiential themes compiled independent of other participants’ data or personal experiential themes.
6. Looking for patterns across participants. Participant personal experiential themes were not compared until all participants’ data were independently analysed through the first four steps. Each participant’s interview transcriptions were completely coded and personal experiential themes compiled separately before moving to this step. This is the step in which all personal experiential themes developed in each of the participant’s interviews were compared to each other. These comparisons led to the generation of a list of final themes and subthemes. Final themes were verified by returning to the data as well as incorporating member reflections to ensure participants’ experiences were accurately represented.

Although themes in a single transcript may merit further investigation in another context, the essence of this process was to understand the phenomenon as it is presented among all amputees interviewed and focused on overlaps of themes among participants.

### Quality and trustworthiness

Phenomenological methods such as reflexivity, member checking, and auditing are often among the most highly used procedures to maximize trustworthiness (Flynn & Korcuska, 2018). We used member reflections instead of traditional member checking and incorporated peer debriefing as suggested by Smith & McGannon, 2018) to ensure rigour in interpretative qualitative research. Member reflections are not participant validation of results but an opportunity for further elaboration of findings and feedback from participants in the co-construction of the phenomenon with the researcher. Member reflections were, primarily, incorporated upon completion of study analysis. Participants were contacted via email and provided both a written and a video summary of findings with a request for a phone or Zoom conversation to discuss the findings and participant reactions to them. Two of the six participants chose to engage in discussion related to analysis and findings.

Throughout the study, the research team engaged in peer debriefing (Smith & McGannon, 2018). The role of the peer debriefing process was to challenge the construction of knowledge through critical feedback that encouraged reflection upon, and investigation of, alternative interpretations emerging from the data. The peers were not directly involved in data collection or analysis and aided in probing the thinking around the research process and analysis (Given, 2008). Peer debriefing occurred approximately weekly during data collection and monthly during data analysis and interpretation. The peer groups that provided regular debriefing sessions included a disability studies researcher, disabled scientists, experts in research among marginalized populations, and people with disabilities whose professions were not academic in nature.

An audit trail was developed which included: initial notes; step by step methods for recruitment, data collection and analysis; annotated transcripts; tables of themes that developed; the final report; and all field notes (J.A. Smith et al., 2022; J. Smith et al., 2009). Field notes recorded details of what was seen, heard, thought, and experienced throughout the course of data collection. Field notes were divided into three categories, each maintained in separate files (Groenewald, 2004). The first was observational notes i.e., objective recordings of what happened, who was involved, what activities occurred, and what was seen or heard. The second were theoretical or reflexive notes recorded immediately after the conclusion of each data collection or analysis session. In these notes, the first author documented reflections on personal experiences related to the data, attempts to derive meaning through reflection on the data, and initial ideas concerning the relationship of data with SDT constructs. The final file contained research notes that summarizes procedures, events, and study progress.

## Results

The main themes that were developed from interviews with participants, analysis, and member reflection feedback are outlined in Table II. Two of the superordinate themes aligned with constructs present in SDT. The other three themes described facilitators to activity, barriers to activity, and the complex processes associated with being active. These influences on participation in LTPA are not described in SDT and are unrelated to participant descriptions of motivation to be active. Participants’ embodied experiences were described throughout their interviews as ways in which the body remembered movements, protected itself from pain, and became integrated with their prosthesis as an extension of their body, rather than a medical device.

**Table II.** Themes developed through IPA process.

Theme	Subthemes
Motivation^a^	Goal setting related to specific LTPA and, separately, to increase functionality as interim step to LTPA participation (identified regulation)^a^
	Pride and concerns about body image influenced motivation to be active (introjected regulation)^a^
Relatedness^a^	Amputee community offers connection not available in any able-bodied social network
	Relatedness enacted as participation in activities with others, directly and indirectly
Facilitators	Prosthesis fit
	Accessible environments
	Activities with intuitive or minimal adaptation requirements
Barriers	Healthcare/insurance system presents roadblocks for mobility devices, prosthesis components, and knowledgeable care
	Prosthesis causes pain or additional complications
	Fear of falling or risk of injury
Amputee Processes to Being Active	Anticipated effect of LTPA on body is necessary component of planning
	Daily use prosthesis is not conducive to LTPA and must be altered, removed, or adapted
Embodied Experiences	Body unconsciously helps participants perform
	Prosthesis and other mobility devices are described as extensions of body

^a^Indicates themes or subthemes that incorporate an SDT construct

### Motivation

Although participants described varying levels of enjoyment in and integration with personal values associated with their chosen activities, intrinsic motivations and integrated regulation to be active were very personal, and not universal. Expressions of motivation to be active related to pride and body image, and LTPA goal setting, were, however, commonalities among participants. These motivations can be described as introjected and identified regulation, respectively. In Figure 1, these motivations are in the centre of the continuum of motivation.

#### Introjected regulation incorporates body image and pride

Participants experienced both pride and shame related to their physical identity as an amputee. Pride and shame provided ego-centric regulators that increased their motivation to participate in LTPA. Negative body image came across as participants described bodily changes occurring after their amputation. Participants gained weight, struggled with how clothes fit over prostheses, and felt the use of mobility devices beyond protheses increased disability stigma. LTPA provided an outlet for controlling weight and improving function to reduce frequency of mobility device use. Participants were motivated to be active to counter poor self-body image or to amplify positive self-body image. The following quote illustrates the role of ego-centric motivations in LTPA participation:

When my former mother-in-law passed away and I was too embarrassed to go to her funeral because I was fat and on a walker. That was the day that I drew a line in the sand and said, “Girl, you got to get your act together. What, what if your sister dies? You know, you can’t avoid that.” And I was just kind of missing out on life. And I just … I had to start all over. (Tiffany, 6.5 years living with amputation)

**Figure 2.**
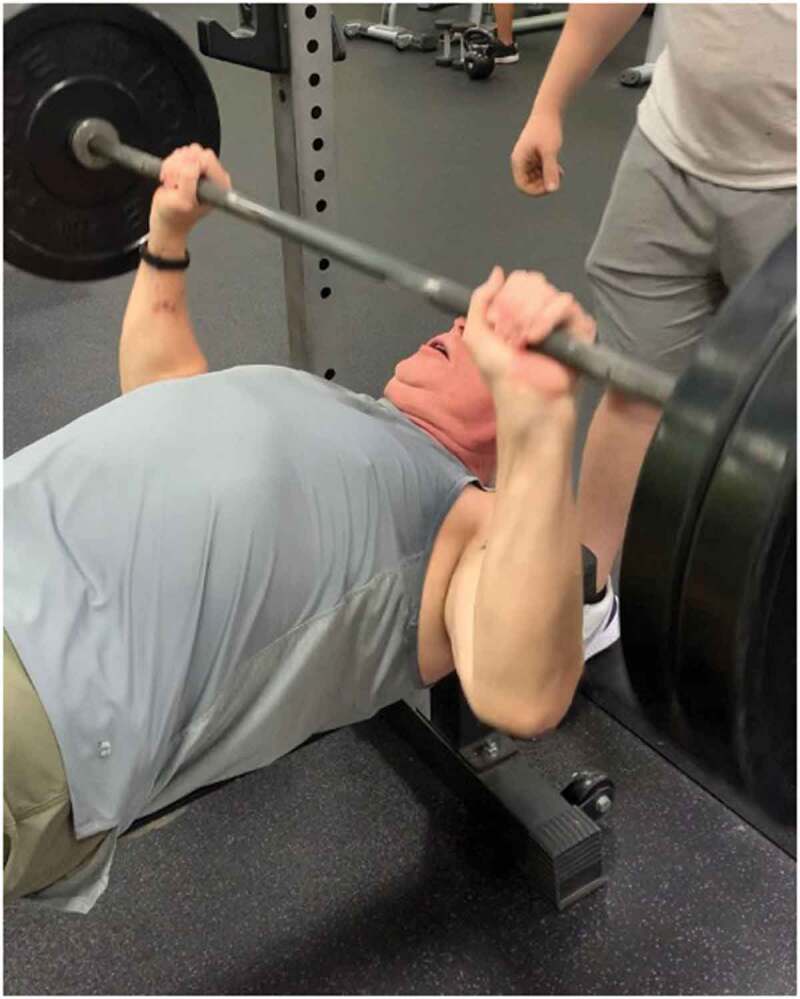
Michael Bench pressing his 5-rep max. Michael felt pride associated with looking good from regular LTPA.

Michael, a 9-year amputee, explained how LTPA also improved how he viewed his body aesthetic (Figure 2). He described being reticent, especially soon after his amputation, to wear shorts, but admitted he liked the reaction he received from being notice for fitness in other areas of his body.

Um, the other thing is, like, um, and I’ll, and this is narcissistic one is when people say, “Wow, you know, um, your, you know, your arms look really good. You’re, you’ve gotten bigger. Your shoulders are bigger, your chest is bigger.” So that’s my narcissistic ones, right? So as much as I, you know, don’t wanna admit that, that’s the other part.

Pride also drove LTPA motivation. Several participants were able to draw on specific moments in which they described pride in their accomplishments. These ranged from familial praise to realizing, after the fact, that they didn’t have to stop and focus before performing a specific activity. Roslyn (6.5 years living with her amputation), described her face-saving in front of her children as a way to become more active and involved in a variety of LTPA after her amputation:

Um, because there’s so much to do and so much to see. Like, like when, before like my accident, like I worked, I took care of my kids. I really didn’t think about much of anything. Like didn’t really put thought into anything. Um, like I- like something like this drastic, it’s, it’s completely life changing. Like overall, like a whole 360. Like there is, my kids have never seen me fail at anything. Um, nor do I ever want them to see me fail at anything. So like my oldest daughter like, I kinda had to like push her out the door. I’m like, “Go.” Like, “You need to go live your life.” I said, “There’s things that I need to do that I might fail at, at first.” I said, “You don’t need to see that.”

#### Identified regulation as goal setting

All participants set goals related to LTPA. Several participants described the need to be able to perform certain functional skills, such as balanced walking using a prosthesis, in order to participate in their LTPA of choice. They set for themselves very specific milestones with the long term goal of becoming (more) active. This did not preclude them from setting specific LTPA goals such as more frequent participation or improved performance in their activity of choice. Dawn, who has been living with her amputation for four years and was bedridden with infection for the first two. After being immobile for so long, she was not initially motivated for LTPA because any level of PA was painful and difficult. While working with a physical therapist, she found activities of daily living (ADL), such as cooking and laundry, to be easier. The pride she felt being able to accomplish ADL for the first time in several years led to interest in LTPA. She described her journey towards bike riding being buoyed by her ability to perform ADL.

Obviously I’m doing this stuff out of necessity, um, but super-duper excited to see and feel the change. I, um, honestly did not think, I, I honestly felt that I was doomed to be on my rear end the rest of my life. And the joy from realizing it don’t have to be that way is tremendous … And every day’s not perfect. There may be a day here or there that it hurts, but I just go in and do, um, additional therapy for myself. And I think that is helping, as a matter of fact, I’m sure it is.

Kristin, a 19-year amputee, used goal setting to motivate herself in the gym:

It’s really easy to get on the thing and just go real slow and you can do that for an hour and, you know, whatever. But if you’re not pushing yourself to go further or faster or- or whatever, every time you don’t, you don’t get the workout that you really need. Um, so that’s the determine … I mean, every time I go in, I’m- I have a goal of what I’m going to do and, you know, and- and try to achieve that.

In both types of goal setting, participants were motivated by the challenge the goal offered. They did not set milestones or goals they knew they could accomplish on the first attempt or even on subsequent attempts after the first success. Instead, participants were eager to challenge themselves to accomplish something difficult, regardless of outcome.

### Relatedness

The most universal and prominent BPN being met among participants was relatedness. Individual participants described competence and autonomy in a wide range of settings, most unrelated to LTPA, but relatedness influenced their motivation to participate in LTPA in multiple ways. The feelings of belonging were generated through LTPA participation, either directly or tangentially through various forms of social support. Feelings of belonging also generated motivation to be active. Connectedness to the amputee community offered a feeling of belonging not available through their able-bodied social networks. This connectedness increased LTPA motivation and participation. Relatedness was enacted as participation in activities with others, both directly and indirectly.

#### Amputee community offers connection

As people with an acquired amputation, participants actively sought out other amputees for a sense of belonging. Fellow amputees provided a degree of connection that only comes from shared lived experiences. The amputee community offered ways to process grief and loss associated with amputation, empathy for physical and mental challenges, and general encouragement. Such connectedness and support extended to LTPA participation. Participants were adamant that this community offered emotional, informational, appraisal, and instrumental LTPA support that able-bodied people did not and were not able to provide. As Roslyn declared, “100%. Like, your family, your friends, they can give you the best support in the world, but they don’t actually know, like, what you’re feeling, but, yeah, what you’re thinking, so yeah. I think that is, like, a must have.”

From a LTPA perspective, relatedness to the amputee community gave access to innovations that enabled activity. Kristin, who doesn’t wear a prosthesis in water, found that her kayak immediately flipped due to the weight imbalance. Her online network of amputees gave her a wide variety of inexpensive ways to adapt kayaking to fit her needs and enable participation. She said,

That’s one of the great things about our community is if you, you can go on any, not any but many of the amputee Facebook pages. And there are several that are athletic inclined and get, you know, 14 different ways people are doing it. And then you can just kind of figure out what is the, the easiest for you.

Participants actively sought out relationships with other amputees through support groups, online forums, healthcare events, and through chance encounters. When a participant’s prosthetist was also an amputee, they expressed greater satisfaction with fit and socket options, driven by two-way trust and the prosthetist’s ability to empathize with the pain and process to perfect fit. In public, participants recognized other amputees and introduced themselves, seeking further connection that may or may not be directly related to LTPA but which drove actions and enhanced feelings of connectedness.

#### Relatedness enacted as participation in activities with others

**Figure 3.**
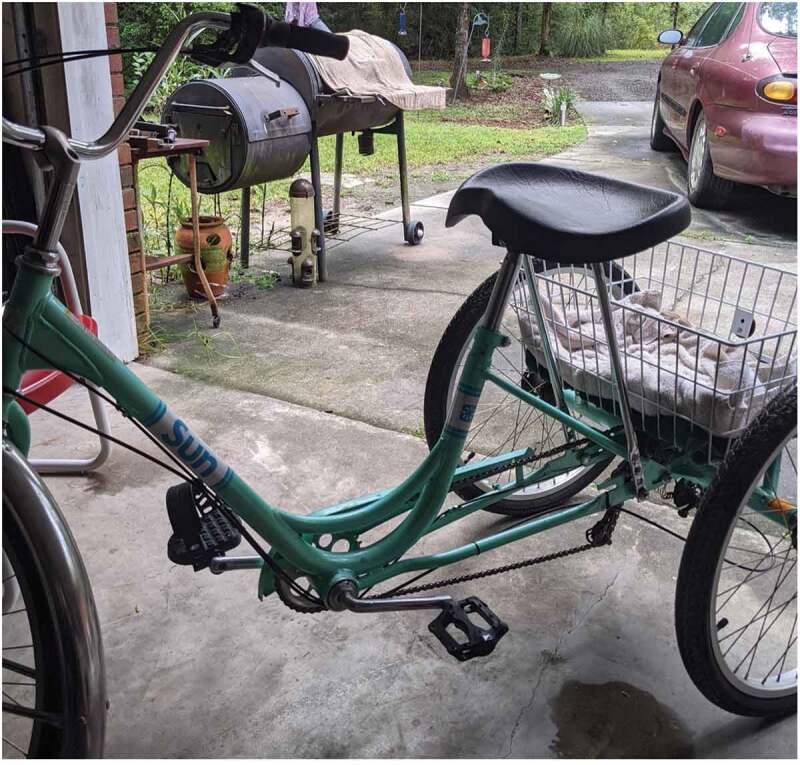
Dawn showing off the basket built for her dog to ride with her while biking. It was important to Dawn to be able to engage in LTPA with her dog.

Throughout each interview with participants, they talked about activities in terms of who else was involved. The involvement could be direct interaction as with Michael’s lifting partners or Summer’s dancing husband; or indirect involvement such as the person Roslyn designed and developed her woodworking projects for. In multiple cases, the who was a dear pet. Dawn’s bike was outfitted with a basket and bedding to allow her dog to ride along (Figure 3). Regardless of who or in what way, being with others was central to LTPA. In Tiffany’s and Summer’s cases the “who” was the impetus for trying a new activity. Tiffany tried seated water skiing for the first time with two other friends who were also amputees attempting the sport for the first time. Summer started Krav Maga lessons based on a family interest,

Sometimes it was having someone there to do it depending on how challenging the concept was. Like Krav Maga I sort of always wanted to do and like I did kickboxing at home, kickboxing videos and stuff, you know, but Krav Maga, my husband, uh, and his son expressed interest too, [he] was the one who instigated.

### Facilitators

Participants identified factors that made LTPA easier. These facilitators, unique to amputees and PwD, did not affect motivation but did support engagement in LTPA once the interest in a particular activity or event arose. These facilitators were: the prosthesis fit, which was heavily influenced by the prosthetist; the accessibility of the built environment surrounding the activity; and the ease at which activities or activity-related equipment were adaptable for participant use.

#### Prosthesis fit

When the socket fit well around the residual limb without slipping, causing pain, or irritating the skin, participants engaged in LTPA more frequently. Prosthetists’ knowledge, experience, and ability to empathize greatly influenced the socket fit. Participants described long journeys to find the right prosthetist, changing healthcare providers multiple times until they felt they were receiving appropriate care. In several cases, one of the deciding factors was the prosthetist was also an amputee. Participants felt an amputee prosthetist was more attuned to the difficulties and pains of an ill-fitting socket and were more aware of advances in technology that would improve fit and function of prostheses, such as microprocessors used in prosthetic knees and socket interfaces with the residual limb. Tiffany’s relationship with her prosthetists have been critical to her function; while Summer only uses an amputee prosthetist for specialized equipment, like her running leg. Tiffany has found that the trust built between her and her prosthetist is anchored in similar lived experiences as an amputee. Her prosthetist is able to use both expertise and personal experience to maximize prosthesis fit, enhancing trust associated with prosthesis adjustments.

My prosthetist now, I don’t know if I told you before or not, but she’s also, and both of my prosthetists, my last two prosthetists have been female and they’ve been AK [above the knee] amputees. But, um, my current prosthetist is congenital. So that’s all she knows, but still, uh, you know, I just, I don’t … I guess that’s, uh … What am I being? Um, what’s the word I’m looking for? I just, I don’t want an able-bodied prosthetist because they can’t relate. They can’t put themselves in my shoes. I want, I want an amputee for my prosthetist because they know what the heck I’m going through, you know. (Tiffany)

Michael, who works in the physical therapy field, was, on the other hand, looking for someone willing to take a person-centred approach to his needs:

So my socket fits well. Um, I have an experimental socket that I developed with my prosthetist and I that’s adjustable with ratchet straps. So it fits tight, snug every day which allows me to do over 10,000 steps a day and to stay at what I would call a high level of exercise for an amputee. Um, at night I take, I put my leg on it around seven o’clock, 7:30 in the morning. I take it off at 10 o’clock at night. It stays on all day. I don’t take it off.

#### Accessible environments

Activities held in environments that were accessible made participation easier. For the participants, an accessible environment was simply about taking into account people with a wide variety of mobility needs. They described even, solid ground, close parking, and event toilet facilities that didn’t require walking through soft ground to access. Although all participants used multiple mobility devices, e.g., wheelchairs, cane, and walkers, most preferred to use their prostheses during LTPA. They found an environment that made mobility less of a challenge encouraged participation in the activity. Dawn was in the process of getting a new socket when we spoke, but was eager to be more active again. She described one of her favourite places to walk,

I just said to my mom’s husband today, um, when we were getting, going to get the pedal for my bike, um, that I wished we could go to [redacted]. It’s a little park, has water, ducks, but all around it has sidewalk with benches and tables and, uh, where I could try to walk. It’s, it’s an even ground.

#### Activities with intuitive or minimal adaptation requirements

Convenience and ease of inclusion into an activity facilitated LTPA participation. As described below, there are additional process considerations with any activity, so for study participants, activities that required minimal additional adaptation made engagement easier. Kristin, a life-long athlete, looked for facilities that would make it easier to get a workout in the gym. She found the recumbent elliptical to be ideal. The rower was too hard to get on an off and stationary bikes required additional equipment, changes to her current prosthesis, or willingness to cycle one-legged. She said, “Um, they have a lot of recumbent equipment because as you get older, that’s just easier for you to do. But when I saw that machine [recumbent elliptical], I told my husband, I said, ‘Oh no, we’re gonna move here because they have my thing.’”

### Barriers

Just as facilitators make participation but did not change motivation level or type, barriers made participation harder, regardless of interest in LTPA. Barriers specific to amputees aiming to be more active included: the healthcare system putting up roadblocks for obtaining appropriate components, devices, and care; prostheses causing pain and other complications when being active; and fear of falling or injury preventing activity.

#### Healthcare/insurance system presents roadblocks

The complex, intertwined healthcare and insurance systems in the country present an overly burdensome obstacle to amputees seeking to maintain health while being active. Prostheses are considered durable medical equipment rather than an extension of an amputee’s body. This lowers the limit on spending. As a result, prostheses that can be used in water as well as for ADL were not approved for Kristin. Dawn waited over two years, using a wheelchair for mobility in an inaccessible home, for her insurance to approve a prosthesis. Summer was required to get a prescription to obtain new liners to protect her skin from rubbing against her prosthesis and causing lesions that prevented prosthesis use both during LTPA and for ADL:

I needed some new liners because the silicone started hardening, so I put in the request with my prosthetist and then a couple weeks later they were like, ‘Oh, by the way, we need you to go get a new prescription and new medical stuff from your GP’ [general practitioner] … So then I had to schedule time, go to my GP, then try and get them to get the paperwork to the prosthetist in the correct way … I’m sitting here with my leg off right now because the liner started hardening, so it’s starts ripping the very sensitive skin.

All participants felt counselling for acquired amputees was limited. Some felt their mental health was not part of their pre- or postoperative engagement. Others experienced ableist comments from providers, such as being told not to consider themselves disabled or that they should not rely on assistive technology as a younger amputee. Most turned to peer and role models found in the community to understand what to expect as an amputee. None of the participants received counselling prior to surgical amputations that addressed expectations of grief or loss, or the physical process and mental tolls for regaining mobility post amputation. This was mentioned as a barrier to becoming active post-amputation, and even identified as a contributor to depression, further reducing interest in LTPA.

When asked what would support amputees in becoming more active, every participant pointed to removal of barriers erected by the healthcare and insurance systems. They felt insurance companies had the power to deny mobility devices based on arbitrary or ableist understanding of how they would be used. One participant, who does not drive, was told she didn’t need a wheelchair that could be easily folded for rideshares because the measure of need for a wheelchair ended at the individual’s place of residence. This limited her ability to plan LTPA activities that required independent transportation. Another was denied a prosthesis with a waterproof knee to participate in watersports because of her age and the insurance company’s assumption that people of her age with limb loss are not active enough to require the device. Michael expressed his frustration knowing he was coming from a place of privilege. By working in physical therapy, in a medical department of a university, he had access to experts and devices that many others do not. He commented,

No matter your financial or social economic condition, that you could get the best prosthesis … And the biggest thing I see mostly is pain from the socket and people think it’s normal and it’s not normal because my prosthesis is so good, I really don’t have any pain at all … So prosthetic fit and be able to afford any, um, prosthetic, uh, items that they can, if they can’t afford, they still can get it. To me that would make people become more active and more social and workout more.

#### Prosthesis causes pain or additional complications

Chronic pain caused by prostheses affected participation in LTPA. Dawn explained that pain associated with her amputation is actually two-fold—the pain from the prosthesis and the pain in her sound leg from compensatory movement.

Being an amputee, there is extra wear and tear on your good parts, your, your good leg, your good knee, uh, your good hip, um, your back. So there’s seems like always, um, something that you’re really having to stop and say, ‘Okay, h- how do I do this with this being like that?’ So it’s a struggle. Every day, honestly, every day is not hard, it depends on how active I am … And it is so painful that in all honesty I have, whether I’m sitting or I’m in my room or whatever, I have been taking it off just for the pain to stop.

Prostheses also cause discomfort during LTPA participation. Michael’s socket becomes very sweaty when biking. This affects the suction and his ability to keep his leg on. There is no pain associated with this but he limits his bike rides to cool weather, evenings, and shorter mileage in order to be able to bike without losing his leg.

#### Fear of falling or risk of injury

As lower-extremity amputees with acquired limb loss, participants had to relearn how to manoeuvre using mobility devices. In general, participants preferred wearing a prosthesis for mobility. Wearing a prosthesis added a layer of relearning to walking, having to balance on the device while manipulating it through movement. Participants expressed concern over falling and associated complications. Dawn was not concerned with the fall so much as her inability to get up off the ground after a fall. Others expressed concern about injury to their anatomical leg that would further limit mobility. Roslyn described a hiking outing with family that nearly wasn’t completed because of the additional care she wanted to take to prevent injury or falls.

And like being an amputee walking on uneven terrain is a challenge within itself, okay? Um, walking on steep inclines, and rocks, and you know, a little stream going through it, with grass. I mean, that was even more of, you know, a challenge. Um, it’s little stuff like that. I mean, and that’s stuff that I grew up doing.

### Process

Participants described multi-layered processes for participating in both ADL and LTPA that, in their opinion, didn’t rise to even a level of awareness for non-amputees. The processes stretched from various methods of self-care and bodily preparation days in advance of an activity to recovery post-activity. Participants anticipated the effect of LTPA on their bodies and prepared accordingly to minimize that effect; they also had to consider their prosthesis functional design and how it may not be conducive to the planned LTPA.

#### LTPA impact on body as component of planning

In addition to general processes associated with daily living that required time and energy not expended by non-amputees, participants described additional processes specific to LTPA engagement. Participants considered the environment they were about to enter and its impact on their mobility and energy level. For instance, Tiffany made sure she had a portable chair for her adaptive waterskiing event because she didn’t know if there would be a long, fatiguing walk from the parking lot to the dock or if there would be any place to rest between ski runs. Summer plans her events in terms of days. If she knows she will be especially active, she stays out of her prosthesis in the day or so leading up to the activity to prevent skin irritation and to build in recovery the day after, making sure she can meet all her work or other tasks in a wheelchair to allow her limb to rest outside of the prosthesis. Dawn said conducting the photo-diary exercise highlighted the extra steps she takes to participate in LTPA such as increasing the number of sock liners used to reduce prothesis slippage while riding, “Um, it’s just showing what I have to do, uh, to bike ride. So I guess the challenges that are faced, uh, as an amputee trying to ride a bike.” Each participant detailed the steps they took, mentally and physically, to prepare for LTPA. They outlined the extra time it took them to prepare, setting out clothes, socket liners and socks the night before an early workout so as not to delay their gym partner; making sure there was a seat set up at the end of a bike ride to be able to recover their shaking leg before putting their prosthesis back on; preparing a space for recovery to minimize movement. Summer described her recovery environment from one of her pictures:

And, um, do I have all my sodas right here? Did I pee, so I don’t have to stand up for three hours? So, so there’s a lot of sort of prep that goes into just being able to sit there … Um, so it looks very calm and chill, uh, but it’s very controlled and planned.

#### Removal or modification of daily use prosthesis to enable participation

**Figure 4.**
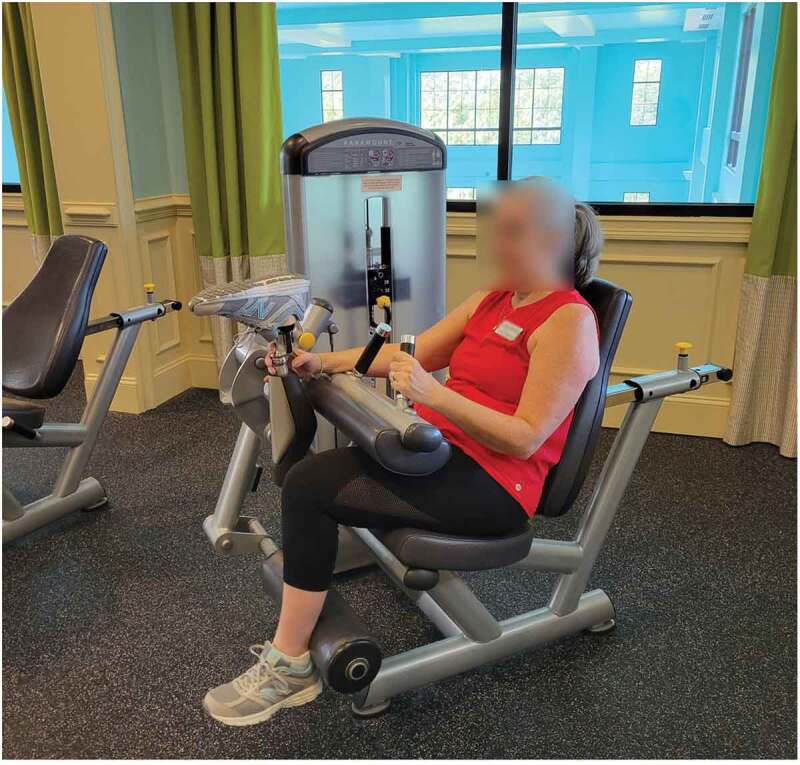
Kristin holding her prosthesis out of the way in order to keep it on while using a hamstring curl machine.

Some amputees have the benefit of multiple prostheses to accommodate multiple types of activities. Most, however, must learn to adapt their everyday leg to an LTPA they wish to do. For some, simply removing the prosthesis is the adaptation that makes the most sense. The weight of the metal and carbon fibre leg become a hindrance to the activity. Roslyn prefers to do most of her woodworking without her prosthesis. She says that after she has prepared her area, much of the physical activity of sanding and carving can be done sitting down, allowing her to leg to rest. Tiffany has found the need to wear orthotics in her anatomical leg’s shoes to add cushioning and protect her knee. She wears the same pad in the shoe on her prosthesis to maintain similar leg length and reduce risk of compensatory injuries. This is a method she discovered on her own after several adjustments to her prosthetic ankle. There is a lot of trial and error associated with determining the modifications necessary to enable safe LTPA. Kristin (Figure 4) used to lift without her prosthesis because it got in the way for some movements. She had to determine her workouts based on whether she was going to wear her leg that day or not. After attempts with different techniques, she was able to adapt her prosthesis to the movement without having to remove it by simply bending the knee up. She talked about her troubleshooting to use the machine,

I tried to do it like a normal person the first time, but that was, like I said … it’s um, just annoying, I guess, for lack of a better word. And so then I said, oh, I can just flip it up and get it out of the way … And so I’ve been doing it like this for 18 years.

### Embodiment

The body and somatic experiences were in the forefront of participants’ descriptions of LTPA. In many cases, the body was primary in how they participated in, reacted to, and experienced LTPA. Embodiment was expressed by some as related to muscle memory, or “being in the zone”, while for others, it was how their body took on the prosthesis as part of them.

#### Body unconsciously helps participants perform

Two ways in which the body improved LTPA performance were a level of focus, often called “being in the zone”, and muscle memory. During interviews, participants enacted a version of muscle memory when describing how they performed certain movements. While talking about the movements, participants performed them, not as a way of demonstrating the movement to the interviewer but as how their body informed recall and articulated the process. Participants also talked about feeling their way into the proper position for lifting or cross-training. They said that it took a long time to determine the best position for their body in a specific movement; that it took professional trainers to watch and correct their movements when they were first learning. But after years of participation, their body let them know if they were in a position of imbalance or potential injury. Kirstin described the full body engagement required for an overhead press, “I can usually tell within the first one or two, because it doesn’t feel the same when I’m pressing down. Um, but that’s from years of doing this.”

“Being in the zone” was highlighted by a detachment from pain and other sensory messages. Michael described a combination of aural focus in which all noise, except his training partner’s voice, was blocked out and his near-constant phantom pain disappeared. Summer had similar experiences in which she did not feel pain while cross-training despite the constant pounding of her prosthesis on her residual limb that would result in abrasions and swelling.

#### Prosthesis and other mobility devices are described as extensions of body

For all participants, their mobility devices, specifically their prostheses, were considered an integrated extension of their own organic body, essentially making them a cyborg being, with both organic and biomechatronic body parts. Michael was very clear in how he embodied his cyborg-ness, “So I do, I am aware of my residual limb. I actually aware of my whole leg, even though I don’t have it from the knee down, but I’m- I’m aware of it.” This connection, not in thought, but in bodily reaction to the prosthesis and LTPA in the prosthesis, helps understand how the body communicates to participants. For Summer, her body reacted to regular workouts, “[by] taking care of myself, then I feel like I can start every step with power, and that’s very healthy for me and my mentality. So largely, I work out because of that.”

## Discussion

**Figure 5.**
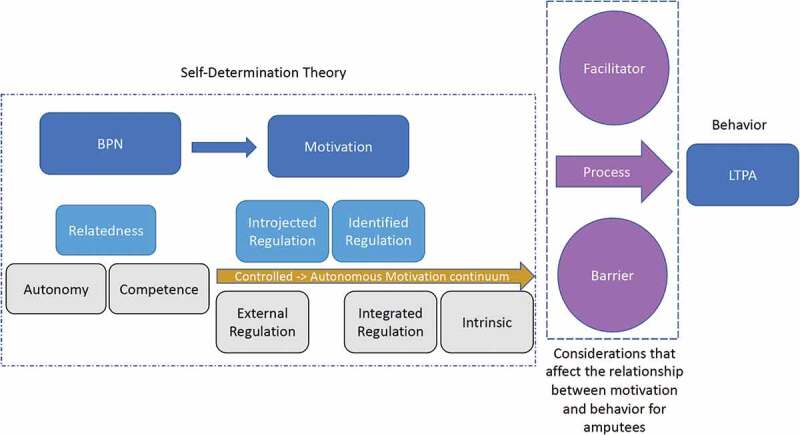
Considerations raised by amputees that impact the relationship between motivation to be active and LTPA participation. The light blue constructs were identified as themes from this study. The purple images represent constructs not addressed by SDT that affect the strength of the relationship between motivation and participation.

This study aimed to better understand amputees’ lived experiences of motivation to be active using the framework of SDT in data collection and analysis. According to SDT, BPN satisfaction supports feelings of intrinsic motivation; motivation then leads to behaviour. Yet, most PA interventions aimed to increase PA participation among PwD do not use a theoretical framework such as SDT to address motivation (Lai et al., 2017; Perreault & Vallerand, 2007). Studies among other populations have shown evidence that SDT partially explains how motivation relates to exercise behaviours, i.e., identified regulation had a positive effect on exercise participation and relatedness was positively associated with exercise (Teixeira et al., 2012; Weman-Josefsson et al., 2015). Participants in the present study experienced relatedness as the most strongly met psychological need related to LTPA. They mentioned their relationship with others repeatedly and with emphasis when discussing LTPA, while autonomy and competence discussions were inconsistent or missing completely from interviews. Participants also described introjected and identified regulations towards LTPA; however, analysis also revealed concepts that influence the relationship between motivation and LTPA not addressed by SDT (Figure 5). Amputees in this study described facilitators and barriers to being active that did not affect their motivation level but did change their activity level. They also discussed the processes required for them to be active that prevented casual participation in LTPA, adding important nuance and contextualization to our understanding of engagement in LTPA. This suggests SDT may not fully explain motivations to be active among amputees, and specifically how those motivations correspond to LTPA participation.

Research in exercise and LTPA with amputees seeks to understand facilitators and barriers to LTPA participation absent an understanding of motivation (Batten et al., 2019; Littman et al., 2014). Alternatively, motivation itself or lack of motivation are identified as facilitators and barriers, respectively (Deans et al., 2012; Lui & Hui, 2009). Current research, however, understanding experiences of motivation through the participants’ perspectives have been absent in those studies. Findings suggests the relationship between motivation, external facilitator and barriers, and participation in LTPA is more complex for amputees than previously understood. The process to become active or recover from activity is also suggested to play a significant role in the relationship between motivation and participation.

Participants’ motivations to be active ranged from experiences of amotivation associated with depression and loss through intrinsic motivations involving pure joy in the activity itself. These extremes of the motivational continuum represented personal experiences that occurred adjacent to the overall phenomenon revealed in this study. Amputees’ collective experiences in motivation to be active were experienced towards the centre of the spectrum—ego-centric motivations and challenge-based motivations—introjected and identified regulations. Participants were motivated to be active to improve their aesthetic or to reduce shame as well as to challenge themselves in LTPA or ADL. These findings are supported by studies interested in specific body image or goal setting outcomes. Holzer et al. (2014) identified amputation as a source of lower body image. LTPA participation improved body image in amputees and generated pride in the way they looked (Galli et al., 2016; Wetterhahn et al., 2002). Inadequate or absent goal setting has been shown to pose a barrier to LTPA among lower extremity amputees (Batten et al., 2019).

Another key finding of this study was the importance of satisfying the BPN of relatedness. As with motivation, participants found unique and personal ways to meet the needs of autonomy and competence, but there was a common experience in satisfying relatedness, specifically the connection to the amputee community in exploring LTPA opportunities. While participants did not necessarily interact with other amputees at any regular interval, they felt a strong bond with other amputees that was not replicable in other relationships. Fellow amputees provided informational support in terms of LTPA adaptations not available through physical therapists or non-amputee activity partners. Amputees traded reviews and recommendations for mobility devices and component upgrades; they supported navigation of the healthcare and insurance systems. Most importantly, fellow amputees provided a deep understanding of lived experiences and emotional journeys that offered a sense of belonging.

### Somatic knowledge as central to experiences of LTPA

Without using the term, participants embodied a cripborg nature (Nelson et al., 2019). Cripborg, in contrast to other cyborg descriptions of disability (see, Howe, 2011; Howe & Silva, 2017; Meyer & Asbrock, 2018), does not equate the bodily integration of technology with an attempt to normalize or superhumanize the disabled body. Howe (2011), for instance, describes the advancements of assistive technology as “help[ing] create a legion of cyborg bodies that is manifest in the image of the sporting supercrip.” Participants used the mobility devices that fit the needs of their activity and their body, moving from prosthesis to wheelchair to walker. Some had preference for their prosthesis but most acknowledged a sort of transmobility (Nelson et al., 2019) created through the use of multiple devices, each an extension of and, fully incorporated with, their body. Merleau-Ponty described the integration of prostheses with the body as more than a physical extension, but one that becomes part of the body’s realm of knowledge, its sensorium (Merleau-Ponty & Smith, 1966).

In addition to their prostheses’ role in embodied cognition, participants discussed embodied experiences of “being in the zone” (*bitz*) during LTPA (Wellard & Pickard, 2017). During times of *bitz*, participants described a sort of detachment from pain and other sensations not directly related to their LTPA. Summer, Roslyn, and Michael frequently described not feeling pain in the moment of activity, despite awareness of what pain the prosthesis, movement, or phantom sensations should or could be eliciting. Wellard and Pickard’s research (Wellard & Pickard, 2017) delve into the complex relationship between physiological, psychological, and social influences that lead to *bitz* experiences and cannot be explained by Cartesian body-mind understandings. In *bitz* there is a sense of being able to close out thoughts and environmental influences to allow enjoyment of LTPA movement; as sort of “going into one’s own world” (Wellard & Pickard, 2017). For lower extremity amputees in this study, *bitz* allowed them to be enveloped in the activity, in both body and mind, without feeling pain, or hearing external distractions.

Shusterman (2011) outlines six types of muscle memory that describes the way in which the body unconsciously influences movement. One of these types, experienced by participants, has to do with the body’s reaction to place. In muscle memory influenced by place, the body’s interactions with location, previous movement patterns, and social environment create a memory of how to move when the place is encountered again. For participants in this study, the fitness environment provided a bodily memory trigger that influenced how LTPA was performed. Participants instinctively knew if their body was in a position to safely and effectively perform the movement required for LTPA and adjusted accordingly.

### Study limitations

The phenomenon being described must be understood as the experiences of motivation to be active among unilateral lower extremity amputees whose amputations were acquired after youth. There are many differences in the lived experiences of lower and upper extremity amputees in how they use, or do not use, prostheses, as well as how those protheses may be integrated into ADL or LTPA. There are also discussions about how embodied prosthesis use is among congenital amputees who do not have the somatic memory of ever having a limb in the place where the prosthesis currently is (Murray, 2008). There may be selection bias in the recruitment of participants. Information about the study described interest in motivation to be active. Participants who were less active may have chosen not to respond to recruitment efforts. All participants in this study were currently active and had some level of motivation to be active beyond external regulation. Experiences of motivation to be active as well as facilitators and barriers to LTPA may be very different for bilateral amputees as it is for unilateral amputees. Additionally body-image is discussed very broadly but may be experienced quite differently for men and women. This study was not large enough to separate experiences by gender.

### Study strengths

Researchers were committed to transparency and rigour. Throughout the development, recruitment, data collection, and data analysis, detailed reflexive and procedural journals were maintained. Member reflections were offered to enable participants’ voices to clearly be heard within the interpretations of the phenomenon. We tried to provide clear descriptions of methodology and study aims to participants and answer any questions they had. The result is a study that has potential impact for policy makers, LTPA interventionists, and researchers. Prior to this study, there were no post-rehabilitative, theory-based qualitative studies done on the lived experiences of amputees with motivations to be active. IPA lends itself to a wide variety of data collection methods, the most common of which are in-depth interviews (Pietkiewicz & Smith, 2014). This study integrated an uncommon method—photo-elicitation—to augment in-depth interviews and add to participants’ opportunities to express their lived experiences of complex and ambiguous topics such as motivation in a variety of mediums.

## Recommendations and future research

Study findings suggest motivations to be active among amputees are influenced by connections and relationships. There is also evidence that the relationship between motivation and participation is not direct, but influenced by facilitators, barriers, and processes undertaken by amputees. The embodied experiences of participants parallel experiences of embodiment described by other researchers, with nuances specific to active lower extremity amputees (Murray, 2008). Each of these have the potential to impact strategies to increase activity among amputees and to expand research with the population for deeper understandings of lived experiences.

### Recommendations for healthcare providers

The complexity of the healthcare system and insurance companies’ arbitrary (from participant’s perspective) determination of what is necessary for ADL worked in conjunction to erect barriers to LTPA at all stages of diagnosis, surgery, recovery, and post-rehabilitation. Simplifying the process and focusing on patient-centred care plans may reduce barriers and lead to better recovery. Acquired amputation, regardless of cause, is a traumatic event. Individuals need to process the grief of loss in addition to focusing on rehabilitation and recovery. Participants, however, did not receive supports prior to surgical amputations to address that loss, nor were supports recommended or provided after the surgery to address the mental processes associated with continued grief amidst the physical recovery to restore mobility. This was mentioned as a barrier to becoming active post-amputation, and even identified as a contributor to depression. Participants were distressed when healthcare professionals discouraged them from taking on a disabled identity or relying on their preferred mobility devices. Rather than embodying the medical model of disability by concentrating on “fixing” a physiological or anatomical malady, healthcare providers have the opportunity to influence longer term mental, physical, and social well-being by acknowledging the roles of social and environmental contexts surrounding amputation (Borowsky et al., 2021). Recommendations offered by participants included mental health counselling before and after amputation procedures, offering amputee peer visitors before surgery, and considering a comprehensive resource list post-surgery of physical and mental health support.

### Recommendations for policy

Recognizing that the prosthesis is not, functionally or emotionally, separate from the body for participants, there is opportunity to integrate mobility devices as part of medical insurance, rather than listing it as a durable medical device. The change in classification, practically, increases coverage amount and may offer more opportunities for mobility devices that enable activity and participation in a wider variety of both LTPA and social activities. Participants also mentioned the built environment as a barrier to participation. Considering paved or solid pathways, including in beach environments, widens the opportunities for people using mobility devices for ADL or LTPA. Policies for new construction, especially of public spaces, should require incorporation of universal design principles, to ensure accessibility and use to the greatest extent possible.

### Future research

The relationship between motivation and participation being influenced by facilitators, barriers, and processes creates opportunity for exploring how SDT may be modified for amputees or other PwD to more accurately reflect the experiences of LTPA and motivations to be active in these populations. The experiences of intrinsic motivation were not part of the overall phenomenon described by participants. Research of the lived experiences of amputees early in their life with limb loss and, separately, after decades of living as an amputee may better describe motivations to be active at various stages in the life-course of an amputee. There may also be differences in the lived experiences of those that identify as being disabled and those that do not. Neither time since amputation or disability identity were explored through this study. Building on embodiment findings, further research is needed on how the body is centred in experiences of amputees beyond LTPA.
